# Coping Strategies Preferred by Patients Treated for Osteoporosis and Analysis of the Difficulties Resulting from the Disease

**DOI:** 10.3390/ijerph19095677

**Published:** 2022-05-06

**Authors:** Agnieszka Barańska, Urszula Religioni, Anna Kłak, Piotr Merks, Magdalena Bogdan, Ewelina Firlej, Anna Sokołowska, Wioleta Kowalska, Bartłomiej Drop

**Affiliations:** 1 Department of Medical Informatics and Statistics with e-Health Lab, Medical University of Lublin, 20-059 Lublin, Poland; bartlomiejdrop@umlub.pl; 2 School of Public Health, Centre of Postgraduate Medical Education of Warsaw, 01-826 Warsaw, Poland; urszula.religioni@gmail.com; 3 Collegium of Business Administration, Warsaw School of Economics, 02-513 Warsaw, Poland; 4 Department of Environmental Hazards Prevention, Allergology and Immunology, Warsaw Medical University, 02-091 Warsaw, Poland; klak.anna2@gmail.com; 5 Faculty of Medicine, Collegium Medicum, Cardinal Stefan Wyszyński University, 01-815 Warsaw, Poland; p.merks@uksw.edu.pl; 6 Department of Social Medicine and Public Health, Warsaw Medical University, 02-007 Warsaw, Poland; mbogdan@wum.edu.pl; 7 Department of Cosmetology and Aesthetic Medicine, Medical University of Lublin, 20-090 Lublin, Poland; ewelinafirlej@umlub.pl (E.F.); anna.sokolowska1@tlen.pl (A.S.); 8 Department of Clinical Immunology, Medical University of Lublin, 20-093 Lublin, Poland; wioleta.kowalska@umlub.pl

**Keywords:** coping strategies, coping with disease, osteoporosis, difficulties resulting from the disease, chronic disease

## Abstract

Osteoporosis has been recognized as a civilization disease. This chronic condition needs a long-term management plan with a holistic approach to patients. The specificity of the patient’s response to the disease and coping strategies are very important in the treatment process. The aim of this research was to analyze the strategies of coping with disease preferred by patients treated for osteoporosis, and to determine the relationship between the self-assessment of patients’ health, time of treatment, sociodemographic variables, and strategies of coping with a chronic disease such as osteoporosis. The study was conducted from August 2016 to July 2018 at an osteoporosis clinic in eastern Poland. Coping Orientations to Problems Experienced (COPE) by C.S. Carver, M. F. Scheier, and J. K. Weintraub in the Polish adaptation and our own questionnaire were used. The study participants were 312 patients treated for osteoporosis. The respondents treated in the osteoporosis clinic used the strategies of seeking support and focusing on emotions to the greatest extent, and avoidance strategies the least. Sociodemographic features and self-assessment of health condition significantly differentiate the strategies of coping with the disease. The analysis showed that the higher the assessment of the individual perception of one’s own health, the more often the respondents used active coping strategies.

## 1. Introduction

Chronic disease is a hard, stressful situation, which mobilizes a person to new attitudes towards the environment. The appearance of these diseases entails long-term consequences for patients, including not only the stress associated with diagnosis and physical pain, but also a number of emotions related to the entire treatment process, often a reduction in the quality of life, and coping with the side effects of therapy [1]. As a result, a patient’s social life and work ability may become significantly limited. Depending on the individual types of behavior, as well as previous experiences, the attitude of the patient towards a difficult situation, such as the disease, may differ [2,3].

Empirical research in the field of health psychology and behavioral medicine has established that the relationship between stress and disease is of mutual dependence [4,5,6]. However, experiencing stress related to chronic illness activates individual specific coping strategies. Learning that one has the disease may be accompanied by a sense of loss of health, or even of threat to one’s life. Chronic diseases require taking two-way actions towards patients. First, professional medical help is necessary, including the treatment process, reducing or eliminating the risk to life, improving the patient’s health condition and their quality of life. The second group of activities necessary in dealing with chronic diseases involves specialized psychological interventions aimed at facilitating the patient’s adaptation to a new, often changed and limiting life situation [7,8]. 

Adaptation to a chronic disease is multi-stage, and begins with experiencing and interpreting the observed symptoms in the context of one’s own needs and limitations. Then, sick people look for help from family, friends, and through the sources available to them or through contact with medical staff as patients [9]. The term ‘coping with the disease’ appeared in the 1960s and was originated by R. Lazarus, who defined ‘coping’ as a stabilizing factor helping to maintain psychosocial adaptation in a stressful situation [10]. The gist of the term is that everyone in the face of a difficult situation produces an individual constellation of cognitive functions, emotional reactions, and a stereotype of behaviors that are relatively persistent, and patients use them at various stages of the disease [11].

Styles and strategies of coping with a difficult situation may be conditioned by cognitive assessment of the disease, which includes knowledge about it and subjective assessment of the future, health condition, and prognosis [12]. Every cognitive assessment causes different behaviors and emotions [13]. Disease treated as an obstacle usually triggers frustration, anger, and an active combative attitude [14,15]. Such emotions may be conducive to coping with the disease, as they prevent the emergence of helplessness, and act as a protective factor against the risk of depression [16]. A disease perceived as difficulty, loss, and impairment causes sadness, resignation, and lowered self-esteem. The threat is accompanied by fear, as well as anxiety [17]. 

The patient’s reaction to the situation of the disease depends to a large extent on individual characteristics and acquired experiences. By conscious or learned modification of the coping strategy, it is possible to change the patient’s perception of the disease itself, and thus support the recovery process [18]. The essence of beneficial strategies lies in implementing reasonable and objective explanations and responses, and focusing attention on the positive areas of the patient’s life [19]. Non-adaptive methods reduce anxiety only temporarily, and unfortunately increase it in the long-term [20,21].

The employed coping strategies are a defense for the organism against stress, which is particularly relevant in the context of chronic illness. The occurrence of the disease generates stressful situations, which induces emotional states that overlap with the clinical picture of the disease, often becoming a secondary risk factor [22]. 

Osteoporosis has been recognized as a civilization disease, and is one of the most common diseases in the adult population [23]. Epidemiological data indicate that osteoporosis is one of the most common osteopathies, affecting approximately 75 million people in Europe, the USA, and Japan, including one in three postmenopausal women, and the majority of elderly people over the age of 70 [24]. It is estimated that in all European Union countries, the prevalence of osteoporosis in women and men aged over 50 is 20–25% and ~6%, respectively [25,26]. A further increase in the incidence is forecasted, due to the progressive increase in the number of elderly people in the world. The gradually increasing number of cases of osteoporosis means that the disease is a serious problem for the health of society, which may, consequently, lead to physical disability and higher mortality. Considering the perspective of an aging society, it becomes particularly important to monitor not only the epidemiological situation of osteoporosis, but also to take measures to prevent the disease and raise health awareness [27,28].

The treatment of osteoporosis should be directed primarily towards the secondary prevention of fractures [29]. The most widely used osteoporosis medications include anti-resorptive therapies: bisphosphonates and the inhibitor, denosumab, which increase bone mineral density and reduce the risk of fractures [30,31]. Other treatments for osteoporosis include estrogen agonists/antagonists, parathyroid hormone analogues, and calcitonin [32]. Although appropriate treatment with medication is important, osteoporosis is preventable with proper management of diet, lifestyle, and fall prevention interventions [33].

The aim of the study is an analysis of the strategies of coping with disease preferred by patients treated for osteoporosis, and determining the relationship between the self-assessment of patients’ health, time of treatment, sociodemographic variables, and strategies of coping with a chronic disease such as osteoporosis.

## 2. Materials and Methods

The study was conducted from August 2016 to July 2018 at an osteoporosis clinic in eastern Poland. The study participants were 312 patients treated for osteoporosis, including 286 women and 26 men. The research included persons treated for osteoporosis for at least one year, who were over 45 years of age, and who consented to participate in the study. These 3 factors were the criteria for inclusion in the anonymous study. The research method was a diagnostic survey in which two questionnaires were used.

The first research tool was the Coping Orientations to Problems Experienced (COPE) questionnaire by C.S. Carver, M. F. Scheier, and J. K. Weintraub, from the University of Miami in the Polish adaptation by Z. Juczyński and N. Ogińska-Bulik [34]. The tool allows for determining the intensity of the general belief of an individual as to the effectiveness of coping with difficult situations, including the disease. The tool measures active, avoidance, cognitive, and behavioral strategies [34]. It is based on a self-report, and consists of 60 statements that are part of 15 strategies for coping with difficult situations. The factor analysis of the tool made it possible to distinguish three factors that explain 77% of the variance: active coping, seeking support and focusing on emotions, and avoidance behaviors. Individual strategies are grouped to form scales according to the COPE tool key, called ‘general strategies’. The result of each strategy was expressed on a scale of 4–16: the higher the value, the more the strategy was used by the respondents. 

The second used tool was our own questionnaire, which supplemented the difficulties related to the occurrence of osteoporosis, the assessment of support, self-assessment of health condition, and sociodemographic data.

### 2.1. Ethical Issues 

The study was conducted in accordance with the human research principles in the Helsinki Declaration after obtaining the consent of the Bioethics Committee of the Medical University of Lublin, Poland, confirmed by the decision number: KE-0254/175/2016. The distribution of the questionnaires to patients was preceded by the Information on the Scientific Research and a request to sign the Informed Consent to Research Form. All respondents were informed about the purpose, as well as anonymity of the research, and gave their consent in writing.

### 2.2. Statistical Analysis

Analysis of the research results was performed in the program Statistica v. 13 (Kraków, Poland). A *p*-value of <0.05 defined the statistical significance of differences. The statistical power was 80% (0.80). In order to determine the relationship between the variables, the following tests and statistical coefficients were used: Mann–Whitney U test, analysis of variance (ANOVA), Tukey’s multiple comparison test, Spearman’s rho, and Student’s t-test. Data of strategies of coping with disease as measured by COPE were analyzed using descriptive statistics (mean, standard deviation, median, max, min). The outcomes of the overall strategies of the COPE tool were calculated by summing the comprised strategies and dividing this sum by the number of strategies. Cronbach’s alpha coefficients for individual scales ranged from 0.48 to 0.94.

## 3. Results

The age of the respondents ranged from 45 to 88 years. The mean age of the study group was 62.76 years. The standard deviation (9.13) establishes a moderate differentiation of the examined people in terms of age. The vast majority of respondents were women (91.7%). The study group was also dominated by persons with vocational education (27.6%), secondary education (25%), and higher education (22.1%). In turn, 15.7% of all respondents had post-secondary education, and 9.6% had primary or lower secondary education. An analysis of place of residence showed that the majority of study participants lived in the city (61.5%), whereas 38.5% domiciled in villages. The vast majority of respondents (71.5%) assessed their financial situation as good, and 11.9% as very good. Only few respondents indicated bad (15.7%) or very bad (1%) financial situations.

The COPE tool measures active, avoidance, cognitive, and behavioral strategies. It consists of 60 statements that are part of 15 strategies for coping with difficult situations. The result of each strategy was expressed on a scale of 4–16: the higher the value, the more the strategy was used by the respondents [34]. The conducted analysis shows that the respondents used general strategies of seeking support and focusing on emotions to the greatest extent (M = 11.3, Me = 11.75), and general avoidance strategies the least (M = 7.58, Me = 7.33). Considering individual strategies separately, it can be concluded that the respondents applied to the greatest extent: turning to religion (M = 12.4, Me = 13) and seeking instrumental support (M = 12.24, Me = 12). The patients practiced the least: use of alcohol or other psychoactive substances (M = 5.23, Me = 4), sense of humor (M = 5.8, Me = 5), and denial (M = 6.56, Me = 6), (Table 1).

The vast majority of respondents (94.9%) experienced muscle and bone pain. More than half of the respondents (60.3%) suffered from limited mobility, and almost half (44.9%) had bone fractures with minor injuries. Reduced body posture was a complaint reported by 27.6% of patients in the osteoporosis clinic participating in the study. One of the respondents indicated that he suffers from joint pain (0.3%). Slightly over half of the respondents (56.4%) assessed their own health condition as good. However, a significant percentage of patients (38.5%) assessed it as bad. Only a few study participants indicated that their health condition is very good (3.8%) or very bad (1.3%). Of note: self-assessment of the state of health is correlated with the employment of disease coping strategies. In this study, our results indicate that the better the self-assessment of the health condition of the respondents, the more they used: active coping (rho = 0.273, *p* = 0.000), planning (rho = 0.265, *p* = 0.000), seeking instrumental support (rho = 0.215, *p* = 0.000), suppression of competing activities (rho = 0.238, *p* = 0.000), acceptance (rho = 0.113, *p* = 0.046), and general strategies for active coping (rho = 0.299, *p* = 0.000), as well as general strategies of seeking support and focusing on emotions (rho = 0.114, *p* = 0.043). Furthermore, the better the self-assessment of the health condition of the respondents, the less they applied the strategy of denial (rho = −0.145, *p* = 0.011), (Table 2).

The analysis of the correlation between the time of treatment of osteoporosis and strategies of coping with a difficult situation showed that the longer the duration of osteoporosis treatment, the more the strategy of turning to religion was practiced by the respondents (rho = 0.119, *p* = 0.036). The longer the treatment period, the less the following strategies were employed: active coping (rho = −0.112, *p* = 0.048), planning (rho = −0.143, *p* = 0.011), seeking instrumental support (rho = −0.158, *p* = 0.005), positive reevaluation and growth (rho = −0.174, *p* = 0.002), as well as general active coping strategies (rho = −0.126, *p* = 0.025) (Table 3).

Almost half of the respondents (46.2%), when asked if it was difficult for them to come to terms with the information about osteoporosis, declared that they had had a hard time only for a few days. However, every third respondent had not been able to accept this information for a long time (32.1%). Moreover, 13.5% of all subjects accepted the diagnosis calmly and without problems, and up to now, 7.7% of all respondents are unable to accept it. Only 0.6% of the respondents did not care at all about this information. When analyzing the limitations associated with the disease, slightly more than half of the respondents (55.1%) indicated that it was difficult to adapt. Furthermore, a significant percentage of the respondents (38.5%) declared that they had difficulties with adapting or not, depending on how they felt during the day, and only 6.4% of all patients did not have a problem with it. 

Difficulties with adapting to the limitations associated with the disease significantly differentiate some coping strategies and one of the general strategies. People who have difficulty adapting to the limitations caused by the disease, to a significantly greater extent than those who have problems or not, applied the strategies of seeking emotional support (F = 3.834, *p* = 0.023), turning to religion (F = 7.863, *p* = 0.000), and general strategies for support and concentration on emotions (F = 5.907, *p* = 0.003); however, they also employed alcohol or other psychoactive substances to a significantly lesser extent (F = 6.537, *p* = 0.002).

Almost half of all respondents (48.7%) indicated family members as those who helped them to accept the disease. Every fifth respondent (20.5%) received the biggest help from acquaintances. For 17.3% of all study participants, the most helpful were friends; for 1.9%, it was a psychologist; and 11.2% declared that nobody supported them. The research showed that active coping (F = 2.927, *p* = 0.034) and suppression of competing activities (F = 3.815, *p* = 0.010) were, to a significantly higher extent, practiced by persons who got help from their family in accepting the disease than people who did not receive any support. When analyzing denial (F = 3.420, *p* = 0.018) and general avoidance strategies (F = 5.215, *p* = 0.002), these strategies were significantly less employed by study participants who got help from their family in accepting the disease than by people who did not receive any support. The strategy of restraint from acting, to a much greater extent, was applied by persons who were helped by acquaintances than by those who had no help (F = 2.845, *p* = 0.038). Planning (F = 4.773, *p* = 0.003) and turning to religion (F = 3.830, *p* = 0.010) were more often used by respondents who were helped by family and acquaintances than by those who did not receive any help in accepting the disease.

Strategies such as seeking instrumental support (F = 13.268, *p* = 0.000), positive reevaluation and growth (F = 3.832, *p* = 0.010), and general strategies for active coping (F = 6.073, *p* = 0.001), as well as strategies for support and concentration on emotions (F = 15.953, *p* = 0.000), to a much greater extent, were applied by persons who received help to accept the disease from someone than by those who had no such help. Conversely, however, the use of alcohol or other psychoactive substances (F = 10.378, *p* = 0.000) and sense of humor (F = 9.053, *p* = 0.000) were significantly more frequently employed by persons without any help than those with some help in accepting the illness. Seeking emotional support (F = 18.991, *p* = 0.000), to a much greater extent, was practiced by persons who received help to accept the disease from someone than by those who had no help, as well as persons who had help from their family compared to those who had help from acquaintances. Analyzing concentrating on emotions and their discharge (F = 6.986, *p* = 0.000) demonstrated that this strategy was utilized, to a much greater extent, by persons who received help to accept the disease from someone than by those who had no such help, as well as respondents who got help from friends as opposed to those who got help from family (Table 4).

### Analysis of the Impact of Sociodemographic Data on the Strategies of Coping with the Disease

The conducted analysis showed that the higher the age of the respondents, the more they applied: turning to religion (R = 0.122, *p* = 0.031), denial (R = 0.198, *p* = 0.000), distraction (R = 0.181, *p* = 0.001), behavioral disengagement (R = 0.171, *p* = 0.002), and general avoidance strategies (R = 0.182, *p* = 0.001). In turn, the higher the age, the less the following strategies were employed: active coping (R = −0.247, *p* = 0.000), planning (R = −0.299, *p* = 0.000), seeking instrumental support (R = −0.317, *p* = 0.000), seeking emotional support (R = −0.254, *p* = 0.000), suppression of competing activities (R = −0.201, *p* = 0.000), positive reevaluation and growth (R = −0.271, *p* = 0.000), restraint from acting (R = −0.167, *p* = 0.003), and general strategies for active coping (R = −0.316, *p* = 0.000), as well as general strategies for support and concentration on emotions (R = 0.167, *p* = 0.003).

The analysis shows that sex differentiates the strategies of coping with the disease. Women, to a significantly greater extent than men, utilized: seeking instrumental support (t = 4.902, *p* = 0.000), seeking emotional support (t = 5.673, *p* = 0.000), turning to religion (t = 4.285, *p* = 0.000), positive reevaluation and growth (t = 2.330, *p* = 0.020), concentrating on emotions and their discharge (t = 3.248, *p* = 0.001) and general strategies for support and concentration on emotions (t = 6.325, *p* = 0.000). Men, in contrast, to a significantly higher extent than women, applied: acceptance (t = −2.234, *p* = 0.026), use of alcohol or other psychoactive substances (t = −8.352, *p* = 0.000), sense of humor (t = −7.245, *p* = 0.000), and general avoidance strategies (t = −6.229, *p* = 0.000).

The analysis shows that the higher the education of the respondents, the more the following strategies were enacted: active coping (rho= 0.123, *p* = 0.029), planning (rho = 0.207, *p* = 0.000), seeking instrumental support (rho = 0.280, *p* = 0.000), seeking emotional support (rho = 0.263, *p* = 0.000), suppression of competing activities (rho = 0.133, *p* = 0.019), positive reevaluation and growth (rho = 0.269, *p* = 0.000), and general strategies for active coping (rho = 0.227, *p* = 0.000), as well as general strategies for support and concentration on emotions (rho = 0.193, *p* = 0.001). In turn, the higher the education of the respondents, the less the following strategies were practiced: denial (rho = −0.264, *p* = 0.000), distraction (rho = −0.188, *p* = 0.001), behavioral disengagement (rho = −0.203, *p* = 0.000), use of alcohol or other psychoactive substances (rho = 0.136, *p* = 0.016), sense of humor (rho = −0.239, *p* = 0.000), as well as general avoidance strategies (rho = −0.267, *p* = 0.000).

When analyzing the financial situation of the respondents, the survey shows that the better the financial situation of the respondents, the more the following strategies were employed: active coping (rho = 0.188, *p* = 0.001), planning (rho = 0.191, *p* = 0.001), seeking instrumental support (rho = 0.240, *p* = 0.000), seeking emotional support (rho = 0.130, *p* = 0.022), suppression of competing activities (rho = 0.164, *p* = 0.004), positive reevaluation and growth (rho = 0.278, *p* = 0.000), and general strategies for active coping (rho = 0.232, *p* = 0.000), as well as general strategies for support and concentration on emotions (rho = 0.127, *p* = 0.025). In turn, the better the financial situation of the respondents, the less turning to religion was undertaken (rho = −0.115, *p* = 0.042).

Place of residence also differentiates strategies for coping with the disease. The inhabitants of the villages, to a much greater extent than the inhabitants of the cities, practiced behavioral disengagement (t = −2.663, *p* = 0.008), use of alcohol or other psychoactive substances (t = −2.588, *p* = 0.010), sense of humor (t = −2.586, *p* = 0.010), and general avoidance strategies (t = −3.065, *p* = 0.002).

## 4. Discussion

Our work demonstrated that chronic diseases such as osteoporosis involve a complex set of stressors, and their consequences in the form of pain or fractures may be the cause of many adverse changes within almost all areas of the patient’s life [35,36]. This statement is in agreement with the outcome of a study conducted among 115 women by Roberto et al., in which an analysis of the relationship between osteoporosis and stress was undertaken. Therein, the respondents declared that they have experienced much greater stress in their lives since they were diagnosed with osteoporosis, and the reasons for the increased feeling of stress were pain, loss of previous roles, and other limitations [37].

The study conducted by Byra among 129 people with spinal cord injury using the COPE questionnaire reveals, as in this study, that the most preferred coping strategies for dealing with a difficult situation were turning to religion (M = 3.64), concentration on emotions (M = 3.02), and seeking support (M = 2.99) [38]. The strategy that the respondents used quite often in Byra’s study was a humorous approach to the situation (M = 3.17), which was applied the least frequently in our own study (M = 5.8). Research with the use of the COPE tool carried out by Mc Hugh R. et al. [39] concerning coping styles in adults with cystic fibrosis indicates that active coping was linked to better social life quality, whereas a negative association was reported between distraction coping with both emotional and social domains. Our own studies lead to similar conclusions, but their analysis covers the aspect of remedial activities when the disease is accompanied by fewer negative emotions, which is indicated by better self-assessment of the state of health. Overall, the better the self-esteem, the more that active coping was practiced.

Bayles et al., summarizing the research conducted, characterized osteoporosis as a disease that devastates the organism not only physically, but also mentally. The authors indicate that the improvement of well-being and the perception of health condition can be achieved through psychosocial support and specific intervention programs in the field of social support addressed to people suffering from osteoporosis [40]. The patient’s attitude and perception of the disease, as well as its acceptance are conditioned, as noticed by Schneiderman et al., by certain personal resources that determine individual differences in the response to the stress associated with the disease [41]. Our own research has shown that the following strategies of coping with a disease such as osteoporosis are applied: seeking instrumental support, positive reevaluation, and growth, as well as general strategies of active coping, strategies of seeking support, and focusing on emotions. The last three are used to a greater extent by people who have been supported in accepting the disease than by people who were not helped by anyone. The opposite trend was related to the strategies of using alcohol or other psychoactive substances and sense of humor, which were applied to a significantly greater extent by people who were not helped by anyone. In order to show the important role of support and other psychological factors in the treatment process, it is worth referring to the conclusions of the study by S. Hamadzadeh et al., which included 275 type 1 and 2 diabetic patients. According to the findings, clinical staff, especially nurses, can improve the self-care and adaption among diabetic patients by encouraging them to apply effective coping methods [42].

In our own study, almost half of the respondents declared that for the first days following diagnosis, it was difficult for them to come to terms with information about osteoporosis. Indeed, according to our results, every third respondent had not been able to come to terms with the disease for a long time, and as of the time of this study, 7.7% of all respondents have still not been able to accept it. When it comes to almost half of the patients, there were family members who helped them to accept the disease, and more than half of the respondents had problems with adapting to the limitations related to the disease, whereas 38.5% stated that they felt such limitations or not, depending on their daily mood.

Confirmation for the analysis of our own research, according to which, patients suffering from osteoporosis have problems with accepting the disease, is the study conducted by Pawlikowska-Łagód et al. among women suffering from osteoporosis, which showed that patients accept their disease to an average degree. Moreover, the study results indicate that an older age when osteoporosis is diagnosed lowers the degree of disease acceptance and increases the difficulty of adapting to disease-related problems. Still, the obtained result (27.3 points), according to the interpretation of the Acceptance of Illness Scale (AIS), indicates an average level of disease acceptance [43].

Many researchers have analyzed the quality of life and the assessment of pain associated with the occurrence of osteoporosis. Paolucci et al., based on analyses of pain in osteoporosis, reported that the incidence of chronic pain tends to increase with age, affecting 41% of people aged 65–75 years, 48% of people aged 75–84, and 55% of all people over the age of 85 [44]. In the case of advanced osteoporosis, especially in elderly patients who are accompanied by chronic pain, the disease leads to a gradual loss of independence and the need for long-term care [45]. The condition for effective treatment and rehabilitation is a well-functioning adaptation process to the new situation, the consequence of which is accepting the disease and life with the disease [12].

### Study Limitations 

Despite the results obtained in this analysis, some limitations were found. Because of the assumption of anonymity of the study, we did not have access to the medical documentation of respondents. However, there are other factors that may have influenced the coping strategies of patients with osteoporosis. It could be useful to classify patients considering the severity of osteoporosis, onset of complication, and which therapy they take. These limitations warrant further investigations with regard to the impact of coping strategies on chronic diseases such as osteoporosis and a more detailed analysis of the treatment process.

## 5. Conclusions

The respondents treated in the osteoporosis clinic in which the research was undertaken used the strategies of seeking support and focusing on emotions to the greatest extent, and applied avoidance strategies the least. Stress resulting from a chronic disease, and related ailments and difficulties with adapting to the limitations related to osteoporosis were declared by the vast majority of respondents, and signify the need for support. Sociodemographic features and self-assessment of health condition significantly differentiate the strategies of coping with the disease. The higher the assessment of individual perception of one’s own health, the more often active coping strategies were practiced. Knowing that coping strategies differ by sociodemographic factors and that they are linked to psychosocial factors is of importance in the design of future studies of coping in persons with osteoporosis. Supporting psychological adaptation to chronic diseases such as osteoporosis and active coping may be linked to an improvement to the osteoporosis treatment process, and this knowledge can be useful for medical staff. Studies are needed to test such interventions.

## Figures and Tables

**Table 1:** Descriptive statistics of strategies of coping with disease as measured by COPE.

COPE—Coping Orientations to Problems Experienced	Min	Max	M	Me	SD
Active coping	5.00	16.00	11.06	11.00	2.26
Planning	4.00	16.00	10.29	10.00	2.65
Seeking instrumental support	4.00	16.00	12.24	12.00	3.25
Seeking emotional support	4.00	16.00	11.29	12.00	3.76
Suppression of competing activities	4.00	16.00	9.64	9.00	2.37
Turning to religion	4.00	16.00	12.40	13.00	3.65
Positive reinterpretation and growth	6.00	16.00	11.44	11.50	2.41
Restraint from acting	4.00	16.00	9.67	10.00	2.20
Acceptance	5.00	16.00	11.60	11.00	2.18
Concentrating on emotions and their discharge	4.00	16.00	9.27	9.00	2.26
Denial	4.00	12.00	6.56	6.00	2.09
Distraction	4.00	14.00	8.70	8.00	1.86
Behavioral disengagement	4.00	14.00	7.63	8.00	1.63
Use of alcohol or other psychoactive substances	4.00	15.00	5.23	4.00	2.25
Sense of humor	4.00	14.00	5.80	5.00	2.26
General strategies for active coping	6.00	15.40	10.42	10.20	1.80
General strategies for support and concentration on emotions	4.50	15.75	11.30	11.75	2.43
General avoidance strategies	5.17	12.00	7.58	7.33	1.14

Abbreviations: Min, minimum; Max, maximum; M, mean; Me, median; SD, standard deviation.

**Table 2:** Correlations between self-assessment of the health condition and strategies of coping with disease.

COPE Coping Orientations to Problems Experienced	Self-Assessment of the Health Condition	
	rho	*p*
Active coping	0.273	0.000 *
Planning	0.265	0.000 *
Seeking instrumental support	0.215	0.000 *
Seeking emotional support	0.140	0.013 *
Suppression of competing activities	0.238	0.000 *
Turning to religion	−0.092	0.105
Positive reevaluation and growth	0.236	0.000 *
Restraint from acting	0.084	0.140
Acceptance	0.113	0.046 *
Concentrating on emotions and their discharge	0.027	0.630
Denial	−0.145	0.011 *
Distraction	−0.083	0.145
Behavioral disengagement	−0.107	0.060
Use of alcohol or other psychoactive substances	0.043	0.454
Sense of humor	0.066	0.244
General strategies for active coping	0.299	0.000 *
General strategies for support and concentration on emotions	0.114	0.043 *
General avoidance strategies	−0.018	0.749

* statistically significant.

**Table 3:** Correlations between treatment duration and strategies of coping with disease.

COPE Coping Orientations to Problems Experienced	Duration of Osteoporosis Treatment	
	rho	*p*
Active coping	−0.112	0.048 *
Planning	−0.143	0.011 *
Seeking instrumental support	−0.158	0.005 *
Seeking emotional support	−0.079	0.163
Suppression of competing activities	−0.041	0.475
Turning to religion	0.119	0.036 *
Positive reevaluation and growth	−0.174	0.002 *
Restraint from acting	−0.052	0.360
Acceptance	−0.061	0.285
Concentrating on emotions and their discharge	0.013	0.826
Denial	0.047	0.409
Distraction	0.083	0.144
Behavioral disengagement	0.051	0.370
Use of alcohol or other psychoactive substances	−0.088	0.122
Sense of humor	−0.050	0.380
General strategies for active coping	−0.126	0.025 *
General strategies for support and concentration on emotions	−0.077	0.177
General avoidance strategies	−0.007	0.897

* statistically significant.

**Table 4:** People helping in accepting the disease and the strategies of coping with the disease.

COPE Coping Orientations to Problems Experienced	Was Anyone Helping You to Accept the Disease?								ANOVA		R.I. Tukey’s Test
	Family (1)		Acquaintances (2)		Friends (3)		Nobody Was Helping (4)				
	M	SD	M	SD	M	SD	M	SD	F	*p*	
Active coping	11.24	2.14	11.05	2.38	11.02	2.43	10.00	2.09	2.927	0.034 *	1/4
Planning	10.59	2.57	10.45	2.63	9.96	2.66	8.83	2.44	4.773	0.003 *	1/4, 2/4
Seeking instrumental support	12.96	2.96	11.88	2.71	12.19	3.38	9.37	3.62	13.268	0.000 *	1/4, 2/4 3/4
Seeking emotional support	12.32	3.28	10.75	3.49	11.00	3.79	7.51	3.59	18.991	0.000 *	1/4, 2/4 3/4, 1/2
Suppression of competing activities	9.95	2.24	9.61	2.35	9.44	2.55	8.49	2.57	3.815	0.010 *	1/4
Turning to religion	12.61	3.40	12.92	3.12	12.20	3.97	10.51	4.67	3.830	0.010 *	1/4, 2/4
Positive reevaluation and growth	11.47	2.36	11.72	2.32	11.59	2.36	10.14	2.53	3.832	0.010 *	1/4, 2/4 3/4
Restraint from acting	9.70	2.19	9.91	2.23	9.78	2.17	8.66	1.89	2.845	0.038 *	2/4
Acceptance	11.54	1.98	11.83	2.45	11.57	2.34	11.20	2.17	0.650	0.584	
Concentrating on emotions and their discharge	9.14	2.06	9.41	2.16	10.04	2.48	7.91	2.15	6.986	0.000 *	1/4, 2/4 3/4, 1/3
Denial	6.24	1.83	6.70	2.39	6.91	2.14	7.31	2.25	3.420	0.018 *	1/4
Distraction	8.57	1.80	8.73	1.90	9.07	2.11	8.51	1.36	1.134	0.335	
Behavioral disengagement	7.54	1.56	7.69	1.57	7.81	1.72	7.57	1.87	0.428	0.733	
Use of alcohol or other psychoactive substances	4.93	1.87	5.27	1.90	4.93	1.89	7.14	3.73	10.378	0.000 *	1/4, 2/4 3/4
Sense of humor	5.59	2.03	5.87	2.16	5.31	1.71	7.57	3.25	9.053	0.000 *	1/4, 2/4 3/4
General strategies for active coping	10.59	1.67	10.55	1.83	10.36	1.91	9.22	1.64	6.073	0.001 *	1/4, 2/4 3/4
General strategies for support and concentration on emotions	11.76	2.01	11.24	2.09	11.36	2.56	8.83	3.02	15.953	0.000 *	1/4, 2/4 3/4
General avoidance strategies	7.40	.94	7.68	1.16	7.60	1.03	8.22	1.76	5.215	0.002 *	1/4

* statistically significant. Abbreviations: M, mean; SD, standard deviation.

## Data Availability

Not applicable.
